# Community Structure in Social Networks: Applications for Epidemiological Modelling

**DOI:** 10.1371/journal.pone.0022220

**Published:** 2011-07-18

**Authors:** Stephan Kitchovitch, Pietro Liò

**Affiliations:** Computer Laboratory, University of Cambridge, Cambridge, United Kingdom; King Abdullah University of Science and Technology, Saudi Arabia

## Abstract

During an infectious disease outbreak people will often change their behaviour to reduce their risk of infection. Furthermore, in a given population, the level of perceived risk of infection will vary greatly amongst individuals. The difference in perception could be due to a variety of factors including varying levels of information regarding the pathogen, quality of local healthcare, availability of preventative measures, etc. In this work we argue that we can split a social network, representing a population, into interacting communities with varying levels of awareness of the disease. We construct a theoretical population and study which such communities suffer most of the burden of the disease and how their awareness affects the spread of infection. We aim to gain a better understanding of the effects that community-structured networks and variations in awareness, or risk perception, have on the disease dynamics and to promote more community-resolved modelling in epidemiology.

## Introduction

Historically, epidemic models have largely overlooked the impact that changes in human behaviour can have on the transmission of an infectious disease [1]. In an attempt to reduce their risk of infection, however, individuals may change their behaviour considerably. A recent example of the occurrence of such changes in behaviour is the 2009 H1N1 pandemic: a study on the psychological responses to the 2009 H1N1 virus, found in [2], reported a large reduction in the use of public transport, a high number of flight cancellations and a considerable amount of investment in preventative goods (e.g. masks). Individuals undertaking such precautionary measures may succeed in reducing their susceptibility to the disease and thus potentially reduce the size of an epidemic outbreak. For this reason ignoring changes in human behaviour can have a substantial impact on the accuracy of many models of disease dynamics.

Classical epidemic models represent a population as randomly mixing individuals, assigned to a pre-defined set of compartments according to their disease status [3], [4], e.g. (Susceptible, Exposed, Infected, Recovered). Another approach to epidemic modelling is to use the concept of metapopulations by dividing the population of potential hosts for the disease into a system of spatially separated, heterogeneous populations (a.k.a. patches) [5]. This separation allows the examination of the persistence of the disease as it spreads within and across the sub-populations [6], [7]. An increasingly popular approach is to model the underlying population as a contact network: a graph in which the nodes represent individuals and the edges represent any contact or interaction that is sufficient for the spread of the disease. The number of contacts of a node are referred to as the degree (or connectivity) of that node. Many types of contact network structures have been studied extensively, including random, lattice and small-world networks [8], because they provide a very different environment for the transmission of the pathogen. For example, it has been noted that epidemic spread is rapid and difficult to contain on networks with small-world [9] and scale-free [10] structural properties. The use of contact networks is an individual-based approach which takes into account the underlying social structure, creating a more intuitive and accurate framework for studying disease spread in large heterogeneous populations [11].

The availability and quality of data today, combined with increased computer power, has led to the creation of very detailed models. The agent-based EpiSims simulation tool, introduced by Eubank et al. [12], uses realistic population mobility data to define a set of locations that people visit, as a part of their daily activities, where exposure to the disease may occur. The EpiSimdemics algorithm [13] is capable of simulating epidemics with very good performance on even larger realistic social networks, while the Simdemics environment [14] utilises a ‘synthetic population’ whose demographics are statistically indistinguishable from the census data used to construct it. The authors incorporate aspects of human behaviour which depend on factors such as household size, income, daily activities and reactions to interventions. Other approaches using census data were also adopted in [15] to study the impact of the timing of social distancing interventions on the disease attack rates; and in [16] to examine the impact of other interventions on the disease dynamics. Wu, Riley, Fraser and Leung [17] have also considered another aspect of human behaviour: compliance with suggested interventions, mentioning that the compliance of individuals may be closely related to various demographics and those levels of compliance may vary over the course of the disease outbreak. Some authors have also modelled aspects of human behaviour without applying high-resolution population data. For example, in [18], the authors examine the role of health care workers in spreading infection by considering three groups: general practitioners, health care workers and rest of the population. The impact of the human population's mobility on the disease dynamics has also been considered, both for long (based on airline traffic data) and short distance travel [19]. All of the above models use real world data to attempt to capture the complexity and heterogeneity of human populations and interactions.

Keeling and Eames [8] emphasise that we are often limited by either time or resources in our ability to construct a social network to represent the population. The size of the population may also be an obstacle as more data and computational power would be required for simulating the infectious outbreak. A comparison between the simulation results of an agent-based model and a structured metapopulation model in [20] has demonstrated that they are in good agreement, with the agent-based model giving more detailed information at the expense of requiring larger and more elaborate data sets for the population. Because of the potential difficulty of obtaining such data, a range of theoretical computer-generated networks have been studied in order to gain a better understanding of the link between their structure and the disease dynamics [8]. In this work we aim to study such a computer-generated network: one consisting of communities of varying size and connectivity, as well as different levels of risk aversion to becoming infected. The existing literature has generally overlooked the concept of community structure in social networks, potentially due to the fact that community structure is still an active area of research in physics and computer science. We intend to make the case for more community-resolved modelling in epidemiology by exploring the disease transmission process on a community-structured network. We demonstrate that this type of modelling can allow us to detect how and when an infection is introduced in a community and what role each community plays in the persistence and spread of the pathogen. Such additional information may not be easily obtainable via existing methodologies which do not consider the communities present in a social network separately. The communities considered in this work have no risk perception initially and, hence, take no precautions to reduce their risk of infection. By introducing risk perception we contrast how changes in behaviour could affect the disease dynamics and how the disease spreads between communities with varying levels of awareness. Our results show that modelling a population in terms of communities could help identifying which groups of people are highly at risk of infection and in studying the different prevalence of the disease in a range of social groups. We also introduce a mean field model to estimate mathematically both the transmissions within and between communities.

In the next section we introduce in detail our definitions of community structure and risk perception. The Methods section describes our algorithm for generating communities that are heterogeneous in terms of size and connectivity and introduces some of the model's concepts and the simulation approach. Our approach to generating communities is novel, although based on an existing algorithm for generating homogeneous communities. The Results section contains our findings and the Discussion section contains comments on potential applications of this type of modelling. In the final section we provide an overview and suggestions for future work.

### Background

#### Community structure

In network theory a community is defined as a sub-network within which there is a larger density of edges between nodes (i.e. internal connections) than there is to any node belonging to a different sub-network (i.e. external connections) [21]. The main focus in community structure research has been designing algorithms for their detection [22]; as a result community structure in contact networks has been widely ignored in the study of the spread of infectious diseases. Girvan and Newman [23] first introduced the community detection algorithm of ‘edge betweenness’ and applied it on many existing networks, demonstrating that identifying community structures can help split both social and biological networks into meaningful clusters. Studies of real world social networks have further revealed that the detected communities are representative of groups of people with highly similar demographics [22].

The concept of metapopulations, described earlier, consists of dividing the population that we are attempting to model geographically, into interacting patches. A similar concept is discussed in [24], where the authors study a small population and propose that larger populations, such as a city, can be modelled as a set of communities that are “in contact through interactions in the work environment or through random interactions in shops or other settings”. We could also choose to divide a target population based on various sociological factors. In our case, we aim to divide the population into communities with different levels of awareness to the risk of becoming infected. For example, access to better healthcare might allow individuals to seek treatment earlier and avoid infecting others. Another example is income: better-off individuals are more likely to invest in preventative measures, thus reducing their risk of infection. Other factors that may cause a higher level of perceived risk of infection could be extensive media coverage, government awareness campaigns, etc. An example of different levels of risk perception is observed in the survey carried out by Goodwyn et al. [2]. In the results the authors observe that Malaysians display more anxiety towards “swine flu” than Europeans and are more likely to take preventative measures. The authors note that the survey results also suggest that people generally perceive pig farmers to be at high risk of infection. Individuals are likely to avoid contact with such ‘high risk’ groups regardless of whether the danger is real or simply prejudice. These and other behavioural observations might be helpful in determining how anxious various individuals are to becoming infected. When considering the common background of the individuals that make up a community, we may be able to use such information to assign the community a level of awareness to the disease using the risk perception framework described below.

In very recent work Gargiulo and Huet [25] have studied opinion dynamics on a community structured network, providing an argument similar to ours: that the population can be split into communities of people of varying opinions. The work demonstrates the benefits of using community structured social networks in modelling the population; however the authors consider opinion dynamics, instead of disease spread, and are mainly concerned with how the network evolves as a response to changes in individual opinions. In this work the network is static: the connections amongst individuals do not vary with time; and we are instead concerned with the impact of risk perception in preventing the spread of infection. We examine randomly generated communities, which are commonly used as a benchmark in the investigation of community structure [22].

#### Risk Perception

We define an individual's risk perception as awareness of the disease based on which he acts to reduce the probability of becoming infected. We model this perception using the framework introduced by Bagnoli, Liò and Sguanci [26], [27]. In this framework, as a result of alertness to the disease, the probability of an individual becoming infected *τ* is multiplied by a factor of
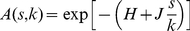
(1)where *s* is the number of the individual's infected connections and *k* is the connectivity (or degree) of the individual. The parameters *J* and *H* represent the individual's awareness. *J* represents individual perception: it determines how strongly the individual reacts to observing the infection in his close contacts. The community awareness parameter *H* determines the awareness that an individual has gained from external factors: media broadcasts, knowledge of adequate precautions, etc. In this study we apply the risk perception approach as a simple framework to represent variations in behaviour between communities.

Studies on risk perception in the social sciences, regarding various hazards, have shown that individuals estimate risk differently depending on the target that is at risk from the hazard. According to empirical observations an individual's perceived estimate of risk tends to be lower when the target is themselves or their families, compared to when the risk target is the rest of the population [28]. The estimates of perceived personal or family risk are likely to increase if the hazard is proximate to the individual. To demonstrate how the model of risk perception above can be representative of real life observations we summarise the results of a recent survey, conducted in Arizona, which examines the risk perception during the 2009 H1N1 pandemic [29] (cited with authors' permission). Analysing the survey results, the authors show that individuals who followed news regarding the pandemic had higher perceived risk of infection, regardless of the risk target. In the framework above this attitude would translate in such individuals having higher *H* parameter than others that do not keep track of news regarding the hazard. Additionally, the authors note that individuals aware of H1N1 cases in their neighbourhood had a higher level of perceived personal or family risk, a phenomenon that could be modelled using the *J* parameter to account for such cases in the individual's vicinity. An interesting finding in this survey is the fact that Hispanic individuals had a higher risk perception than non-Hispanics, which further supports the idea of different levels of risk perception between communities introduced earlier. In fact, if we assume that Hispanic individuals have more Hispanic than non-Hispanic contacts, in this case the sample population can be divided into at least two communities: a non-Hispanic and a Hispanic one, with the latter having higher risk perception. This assumption is not unreasonable considering the homophily (i.e. ‘birds of a feather stick together’) property of real-word social networks, as well as the fact that some of the Hispanic individuals surveyed in [29] spoke only Spanish.

Some recent epidemiology research has revealed increased interest in how awareness of the disease incites people to take measures to reduce their susceptibility. Barrett et al. [14] state in their conclusion the importance of studying the spread of fear or information in response to the epidemic. Funk, Gilad, Watkins and Jansen [30] have already taken a step in this direction by studying two networks simultaneously: one on which the disease spreads and a second one on which information regarding the disease is propagated. Economists have also called for the incorporation of human awareness in existing epidemiological models and have suggested that people's responses are likely to be influenced both by public (in this framework *H*) and private (*J*) information [31]. A risk perception approach has already been applied to the problem of studying individual decisions on getting vaccinated during an epidemic outbreak [32]. An examination of the different effects of risk perception on a scale-free network without any group structure is available in [33].

## Methods

In this study we consider a static network of *N* individuals, which can be completely described by five parameters:

- *C* is the number of communities.

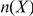
 is the size of community *X*, given as number of nodes.

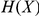
 is the awareness of the disease in community *X*.

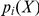
 is the probability that a node in community *X* has a connection with another node in the community *X*.

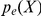
 is the probability that a node in community *X* has a connection to another node that belongs to any other community.

In addition we use 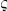 to denote the set of all communities in the network. Four of the parameters above are necessary for the construction of a network consisting of heterogeneous random communities, as they allow for varying sizes and levels of connectivity. The *H(X)* parameter is necessary only when the communities modelled also have varying levels of risk perception. The commonly used planted l-partition model [34], unlike this method, constructs equally sized homogeneous communities.

The large number of parameters makes conducting a detailed study difficult. For this reason we have chosen to keep the number of communities constant, *C = 5*. Using a set of five communities we are able to study a good range of combinations of the remaining four parameters and examine their general effect on the disease spread within and across the communities. In the Results section we also examine how the exposure to disease of a single community is affected by variations in these parameters.

### Network Generation

A common approach to generating networks for testing community detection algorithms is to use the planted l-partition model [22], [34]. The algorithm divides a set of *N* nodes into *l* equally sized groups. Two probabilities are defined:

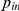
: the probability of a node having a connection to another node in the same group.

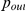
: the probability of a node having a connection to another node from a different group.

Links are generated between all pairs of nodes according to these two probabilities and the result is an Erdös-Rényi-like random network of *l* communities, provided that 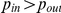. The shortcomings of this method are that the *l* groups are equally sized and that the number of internal and external connections is roughly equal for all individuals in the network. Our approach, described below, generates a network of communities in which the communities do not have to be equally sized and the connectivity of individuals is similar for members of the same community but varies widely between communities.

The generation process is as follows, using the parameters specified earlier:

1. We assign each node to a single community, according to the communities' sizes.
2. For each community *X*:
  1. For every node 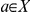:
    - For all other nodes 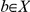: create a directed link from *a* to *b* with probability 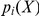.
    - For all nodes 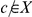: create a directed link from *a* to *c* with probability 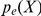.
3. For all pairs of nodes *a* and *b*:
  1. If there exists a directed link both from *a* to *b* and from *b* to *a* then do nothing. Otherwise delete any directed links between *a* and *b*.

**Figure 1:**
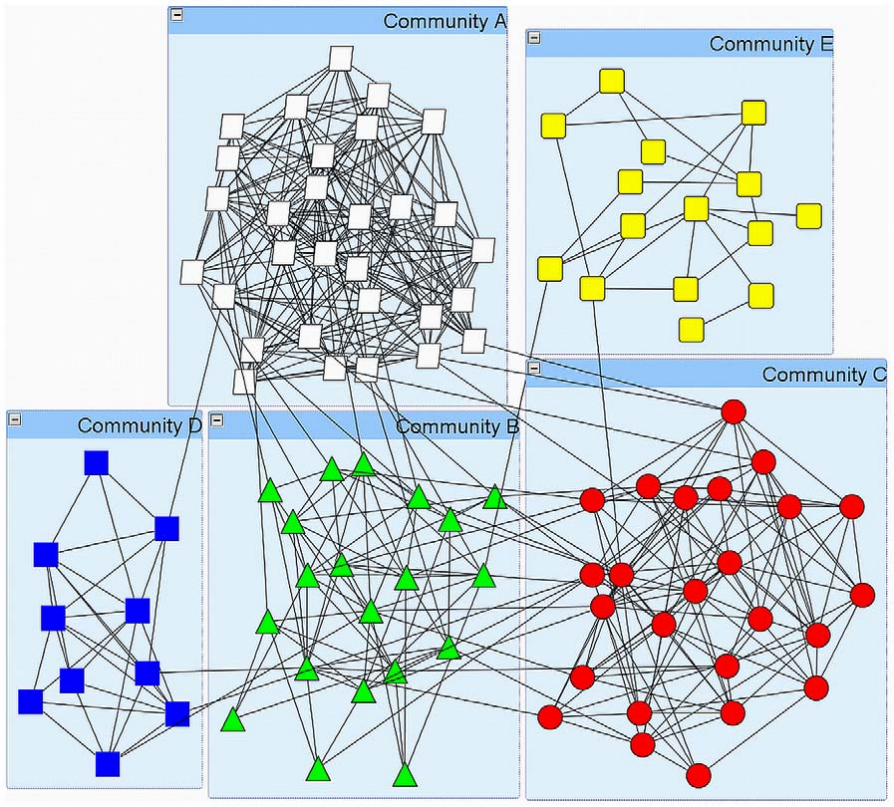
Sample community structure, constructed with the parameters summarised in **Table 1**.

We can use the adjacency matrix M to denote the weight of the connections between all individuals in the population. Since we are already considering a large number of parameters in this paper we have chosen to keep the connection unweighted. In other words:
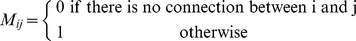
and 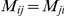. A sample set of parameters is given in Table 1 and the resulting network is shown in Figure 1.

**Table 1:** An example of five heterogeneous communities and their parameters.

Community	A	B	C	D	E
**Number of nodes ** ***n*** ** (given here as % of total population)**	30	20	25	10	15
**Community Awareness ** ***H***	0	4	2	3	1
**Probability of internal contact ** 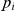	0.7	0.6	0.6	0.8	0.5
**Probability of external contact ** 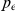	0.1	0.25	0.1	0.05	0.03

The communities resulting from the above algorithm are Erdös-Rényi random graphs and hence have the small-world property: any two nodes within a community are likely to be connected via a small number of intermediate acquaintances. This property also holds across separate communities, although the average number of intermediate nodes is likely to be larger, due to the lower density of edges between communities. More realistic networks would also exhibit clustering, which could also be included in this model by taking into account that if node *a* is connected to both nodes *b* and *c*, then it is more likely that nodes *b* and *c* are also connected to each other. Although such considerations could help in creating a more realistic computer-generated population model, they would substantially complicate both the generation process and the rules presented below.

In the above description of the generation process we have assumed that every node has been already assigned a community in Step 1 and have not discussed any mechanism for determining each community's size. A given community size would be acceptable provided that, together with the community's internal and external connectivities, the definition of a community is not violated. Note that even in the case where all the communities are of equal size the internal and external connection probabilities may still vary between communities, as long as the resulting community still has a higher density of internal than external connections. Below we provide a quantitative definition of a community, linked to the parameters used in this paper, so as to provide a set of rules to adhere to when choosing each community's size and connection probabilities. In [35] two definitions of a community are given. A subset of nodes *V* is a community in the weak sense if
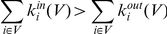
where 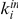 is the number of edges connecting node *i* to other members of the subset *V*, and 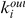 is the number of edges connecting node *i* to nodes belonging to the rest of the network.

Similarly, a subset of nodes *V* is a community in the strong sense if
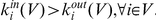
In our case the connectivity *k* depends on the size of the community as well as its probabilities of internal and external contact. With the parameters, given in Table 1, our algorithm will produce a community in the weak sense if, for community 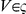 , the following holds:
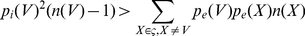
(2)Inequality (2) ensures that the average internal connectivity of any node will be higher than its external connectivity, thus generating community structure.

For a community, generated by our algorithm, to be in the strong sense it is necessary that
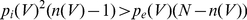
(3)In equation (3) every single node in the community will always have a higher internal than external connectivity, even in the case where, for some other community *X*, the external connectivity 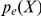 takes the maximum value of 1. All the communities in Table 1 are communities in the strong sense, except for the weak community B. To ensure that a community structure is obtained, the left hand side of either inequality (2) or (3) should always be sufficiently larger than the right hand side. Otherwise, due to the stochasticity of the generation process, the network may not have the required structure on some realisations of the algorithm. Even a small difference is sufficient to ensure that a correct network topology is generated.

The parameters for external and internal connectivity in Table 1 are suitable for fairly small networks. In large networks these parameters may cause some nodes to have exceptionally large degrees. This occurrence is due to the number of nodes both within and outside the community being very large, resulting in many connections being formed. For the transmission of most diseases close contact is necessary and people tend to have only a limited number of such close contacts per day. To account for the limited number of contacts we chose parameters 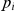 and 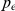, using equations (2) and (3), such that the connectivity of the nodes in each community does not exceed a reasonable upper bound. The communities examined in this paper match the parameters of Table 1, with the probabilities scaled to allow for large size networks.

### Boundary Nodes

Studying the effects of boundary nodes (i.e. nodes within a community with at least one external connection) is a common procedure when examining community structure in networks [22]. In this work boundary nodes represent the only means by which infection can travel between communities.

We can estimate the average number of boundary nodes for the individuals within a community mathematically. In our model, the probability of two nodes, members of communities *V* and *X* respectively, being connected is 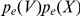. The probability of a node in *V* not having a connection to any node from community *X* is 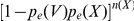. By considering all other communities in 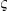 and subtracting from one, we obtain the expected number of boundary nodes for community *V*:
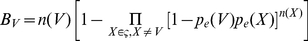
(4)The above result is used below in creating a mean field model approximation, as well as in studying the behaviour of a single community.

### Single Community

In order to examine how the parameters of a community affect its exposure to disease from the outside, we set up a susceptible community connected to a completely infected outside world. Running the network generation algorithm for different values of external connectivity 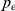 and community size *n*, we obtain the average number of transmissions entering the community per unit time. Parameter values that do not generate a community in at least the weak sense (see equation (2)) are ignored. We repeat this procedure for different values of *H*. Note that, since all individuals in the community are susceptible and the outside world is completely infected, we are not concerned with the value of 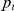: we only observe how infection is introduced from outside the community.

The expected number of infections could also be estimated by calculating the expected number of boundary nodes that would become infected: in a community of susceptible individuals all initial infections would have to be introduced from the outside. The expected number of infections that would occur in this situation is given by
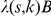
where *B* is the number of boundary nodes that the community has, estimated using equation (4). In this experiment only neighbours outside the community are infected, which means we can reduce 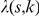 to
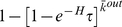
(5)where 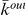 is the average number of external connections 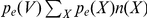. Using the mathematical approximation we obtain estimates for the average number of infections entering the community, which are in very good agreement with the simulation results, presented in the Results section.

### Simulation

In this investigation we implement both the Susceptible-Infected-Susceptible (SIS) and Susceptible-Infected-Recovered (SIR) models. In the SIS model, upon recovery from the disease each individual becomes susceptible to infection again. An infectious individual becomes susceptible again with a probability *γ*, kept constant throughout this study (*γ* = 0.2, expected recovery of five time steps). In the SIR model, on the other hand, an individual does not become susceptible following infection, but rather recovers with a probability *γ*, and can no longer infect other individuals or become infected itself. In general, changing the transmission model is not difficult, and considerations such as asymptomatic infections, occasionally used in modelling influenza, can also be implemented, see the end of this section for a brief outline.

The reason we have chosen to consider the SIS model is that, as susceptibles are reintroduced into the population, the disease may become persistent. As a result, simulating the SIS model would show the prevalence of the disease within a community over long periods of time as individuals become re-infected and the model reaches endemic equilibrium. Using an SIS model also allows the disease to be reintroduced in a community where the infection has previously become extinct and the frequency of these reintroductions can be considered. Furthermore, the overall burden of the disease on each community over a long period of time can be estimated. Despite allowing us to easily examine these phenomena, the SIS model may not be the most realistic one to apply, given our definition of risk perception.

The risk perception framework, defined in (1), is suitable for considering the awareness of the population to an epidemic disease, but it may be unsuitable for considering an endemic disease. If a disease is in endemic steady state then the awareness to the disease is likely to also be dependent on the time period for which the disease has been circulating. For a persistent disease, awareness may actually increase with the time since the disease was first introduced. The risk perception framework does not take such timing into account, and is concerned only with the number of infected individuals, which would be suitable for short epidemics. Since the risk perception framework is based on background material that examines mainly pandemic influenza, the more appropriate SIR model is also considered in this paper. Unlike the SIS model, we cannot examine the fraction of the population infected over a long period of time because the disease is transient, so instead we consider the final size of the epidemic: the total number of individuals infected before the disease becomes extinct.

In the individual-based simulations we assume that the probability of the infection transmitting along a contact link is proportional to the weight of the link 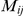, although multiplied by a factor that represents the individual's risk perception (equation (1), reproduced):
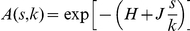
The probability that any susceptible individual becomes infected from one of his infected neighbours is
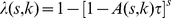
(6)where *τ* is the probability of infecting one of the individual's contacts and is representative of the infectivity of the disease. If the individual is recovered then 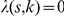 so that re-infection is impossible. To model the fact that, despite belonging to the same community, the awareness may vary somewhat between individuals we also introduce some white noise 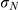 to *H*. The white noise represents *quenched disorder*, as it does not evolve over time, and is used to model the slightly different magnitude of risk perception that an individual may have regarding the disease. The parameter's mean is 0 and it has a variance of 0.1. Those values were chosen so as to affect the individual's personal awareness without overly deviating from the community-wide *H* value.

The probability that an infectious individual infects an acquaintance, *τ*, can be linked to the basic reproduction number 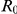: the average number of people infected by a single infectious individual in a completely susceptible population. The basic reproduction number is an important metric in epidemiology due to its threshold property: if 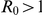 the disease will be able to spread through the population, otherwise the disease will become extinct without causing a large epidemic. The value of 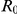 is a combination of the infectivity of the disease and the contact patterns of the individuals in the network. Gross et al. [36] suggest that for random networks
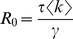
(7)where 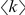 denotes the mean connectivity of the entire population .

To obtain our results, we run a large number of simulations, infecting a small fraction of the population at random at the start of each run. At every time step each node with infected neighbours can become infected with probability 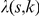 and infected individuals recover with probability *γ*. We construct a new network for every simulation, to obtain data for different network topologies, and average the results. For both the SIS and SIR models we examine the number of transmissions originating from each community. We initially consider the case of no risk perception and identify which of these transmissions are external, i.e. the infection is transmitted to individuals outside the infector's community. We then repeat the study for both models with risk perception introduced, allowing us to examine the role of each community in transmitting the infection and to determine how these roles change in the presence of risk perception. Additionally, the effect of changing the value of the disease infectivity *τ* on the number of transmissions per community is also examined for both models. As mentioned previously, for the SIS model, we also study the prevalence of the disease in each community and the amount of time spent sick per community (which can be used to quantify the burden of the disease on the community) to provide an overview of the disease dynamics over a long period of time. Finally, to demonstrate applications of the model we provide two examples: one treating the risk perception as being the result of mitigation strategies aiming to reduce the impact of the epidemic and a second example examining the time taken for an outbreak in one community to reach the rest of the network.

### Mean Field Analysis

A mean field model aims to reduce the dynamics of a complex system to a mathematical representation of its effective behaviour. In the case of disease spread it is common to reduce the dynamics to a set of ordinary differential equations (ODEs) that describe the system's evolution with time. Constructing a mean-field model of our system allows us to examine and summarise its expected behaviour, and to compare the results with the individual-based simulations. This comparison can be used to confirm the correctness of our implemented simulations. Furthermore, the mean field can be used to estimate the epidemic dynamics, without the need of executing the individual-based model.

#### The SIS Model

Consider the individuals of a community as being divided into two groups: boundary nodes, as determined by equation (4), and the rest of the nodes having no external connections. The non-boundary nodes can only acquire infection from individuals in the same community, whereas boundary nodes can acquire infection from either their own community or the external nodes that they are connected to. The force of infection experienced by any susceptible individual can be defined as
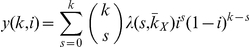
where 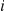 is the fraction of infective individuals, and 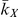 is the mean connectivity of a node in community *X*:
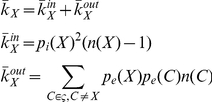
and 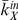 and 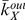 are the node's internal and external only connectivities respectively. Note that we use 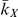 in the definition of 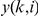 above, so that a correct estimate in the risk perception function in equation (6) is obtained. If there is no awareness and both *H* = 0 and *J* = 0 then 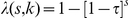 and function 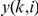 reduces to that of the standard SIS model [37].

Next we define the fraction of infected individuals both within and outside a community *X*. The fraction of infected individuals inside a community is simply
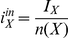
where 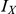 denotes the number of infected individuals in community *X*. The expected fraction of infected external acquaintances of community *X* is given by
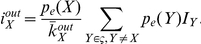



Using these definitions we can write expressions for the force of infection experienced by both boundary and non-boundary nodes. If 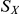 is the number of susceptible individuals in community *X*, then there are

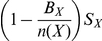
 susceptible non-boundary nodes, experiencing a force of infection of 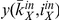

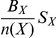
 susceptible boundary nodes which can acquire infection from either outside or inside the community, experiencing a force of infection of 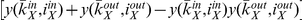

Thus our model, for a community X, can be described using the following ODEs:
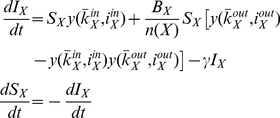
(8)


#### The SIR Model

The SIR model is similar to the one described in (8), with the exception that following infection individuals recover and do not re-join the susceptible class. Thus, although the equation for 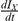 is the same as before now we have an additional recovered class, and the system is described by:
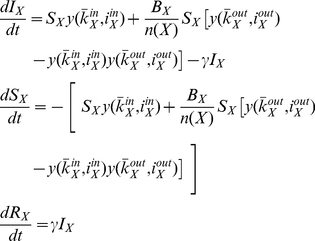
(9)In order to compare the mean field approximations against the simulation results we examine the effect of increasing parameter *J* per community, since it also influences risk perception. We remind the reader that *J* is the personal awareness of an individual: it modulates the amount by which an individual's awareness increases from observing disease symptoms in any close contacts. Initially we run the mean field approximation assuming the communities are isolated, i.e. without accounting for the effect of boundary nodes. Removing the effect of external infectious individuals can be achieved by setting 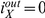 (and hence 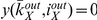) which reduces the rate of change in the number of infective individuals in equations (8) and (9) to
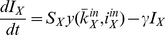
(10)and changing 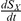 in both models accordingly. We then introduce boundary nodes by applying the original equations (8) and (9) instead and observe the differences in the estimates. We have chosen to approximate only internal transmissions initially as doing so would allow us to evaluate the effect of the boundary nodes on the disease dynamics. We compare the mean field results of both models to the dynamics observed in the simulations.

### Potential Alternative Transmission Models

In the study we use the SIS and SIR models. The SIS model allows us to examine how the infection spreads (and is potentially reintroduced) over time, whereas the SIR model is more applicable to the disease awareness framework that the paper implements. Applying a different transmission model to our framework is also possible. For example, if we wished to implement a Susceptible-Exposed-Infected-Recovered (SEIR) model we could easily do so by defining the mean period of time *∝* for the Exposed period, so that at every time step an individual in the exposed state becomes infected with probability *1/∝*.

An important consideration might be to allow for the modelling of diseases with asymptomatic infectious cases, such as for example Influenza [17]. Here we only briefly describe how such a transmission model can be implemented, as an example for an extension to our framework. Asymptomatic individuals may still be infectious, although potentially less so than symptomatic cases. To model this we introduce a constant 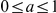, such that if 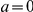 asymptomatic cases are non-infectious and if 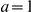 asymptomatic and symptomatic individuals are equally infectious. All other values imply a reduced transmission rate for asymptomatic infections. We need to define the fraction of individuals that will never develop symptoms; for example, in the case of Influenza 1/3rd of cases may be asymptomatic [17]. We also split the number of infected neighbours *s* , so that 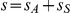, where 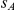 is the number of asymptomatic infected neighbours and 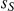 is the number of neighbours who are visibly infected. In this case, the probability of an individual becoming infected (6) is
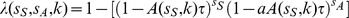
In the presence of asymptomatic infections with 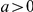 an interesting case for risk perception occurs. An individual may become infected from any of his contacts, regardless of whether their symptoms are visible or not. However, an individual can only be aware of the infection if he observes the symptoms and, thus, his risk perception will only be based on his number of symptomatic contacts 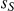. In the presence of asymptomatic cases 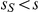, meaning that individuals will underestimate the disease's prevalence in their vicinity and the risk perception level is below optimal. This inefficiency could have a significant effect on the system: if the number of asymptomatic cases is very high then the personal awareness value *J* may have little to no effect in reducing transmissions.

## Results

This section summarises the results obtained from the experiments described in the Methods section. Examination and interpretation of the results can be found in the Discussion.

### Single Community

**Figure 2:**
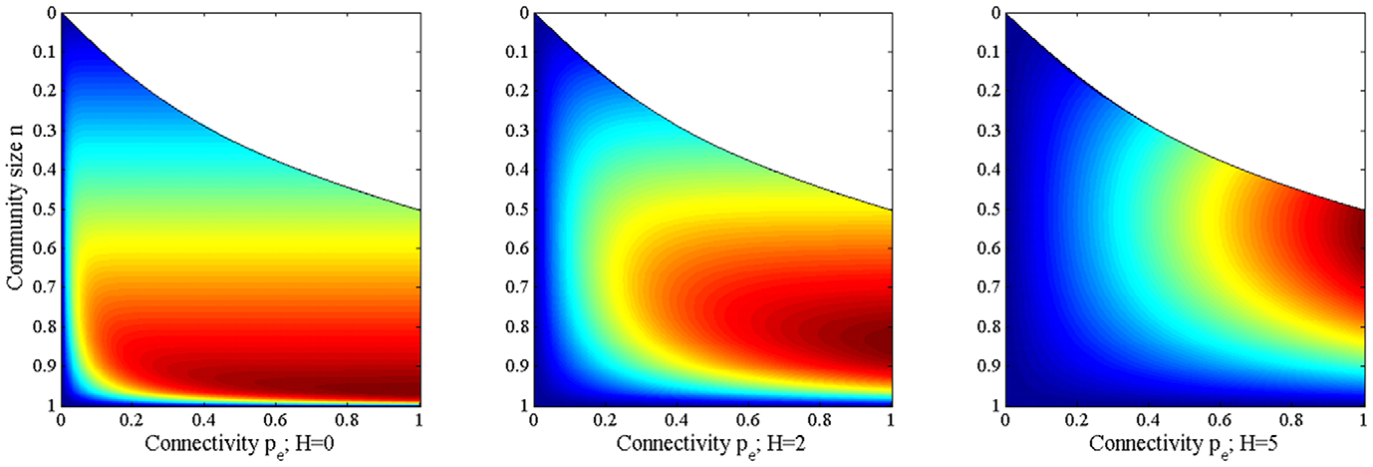
Average number of infections entering a single community for varying community size and connectivity. White areas represent parameter combinations that do not produce a community structure. Community size *n* represents fraction of total population size *N*. Replicated for three values of *H*.

Setting up and running the single community simulations described previously we discover that, in general, very large communities (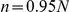 or higher) or those with very low 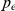 experience the least exposure to infection from the outside. This result can be seen in Figure 2 and is consistent for all values of *H*. In Figure 2 we can also see that, as we increase *H*, the parameters of the most highly exposed community shift. In the case of no risk perception we observe the most infections in large communities with medium to high 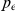. As *H* increases the highest number of infections is instead observed in medium sized communities with high connectivity.

The above study only examined the process of an infection entering a community from the outside, and not its subsequent spread within the community. An isolated community in our case is simply an Erdös-Rényi random graph, and the spread of disease within these graphs has already been studied in detail [8]. Note that, since we are estimating the number of infections entering a completely susceptible community, the result is equivalent for both the SIS and SIR epidemic models.

### Mean Field Analysis

In the following results we have used higher values of *τ* than in the rest of the Results section, so that the disease prevalence would also be noticeably high in the communities with high levels of awareness.

#### The SIS Model

**Figure 3:**
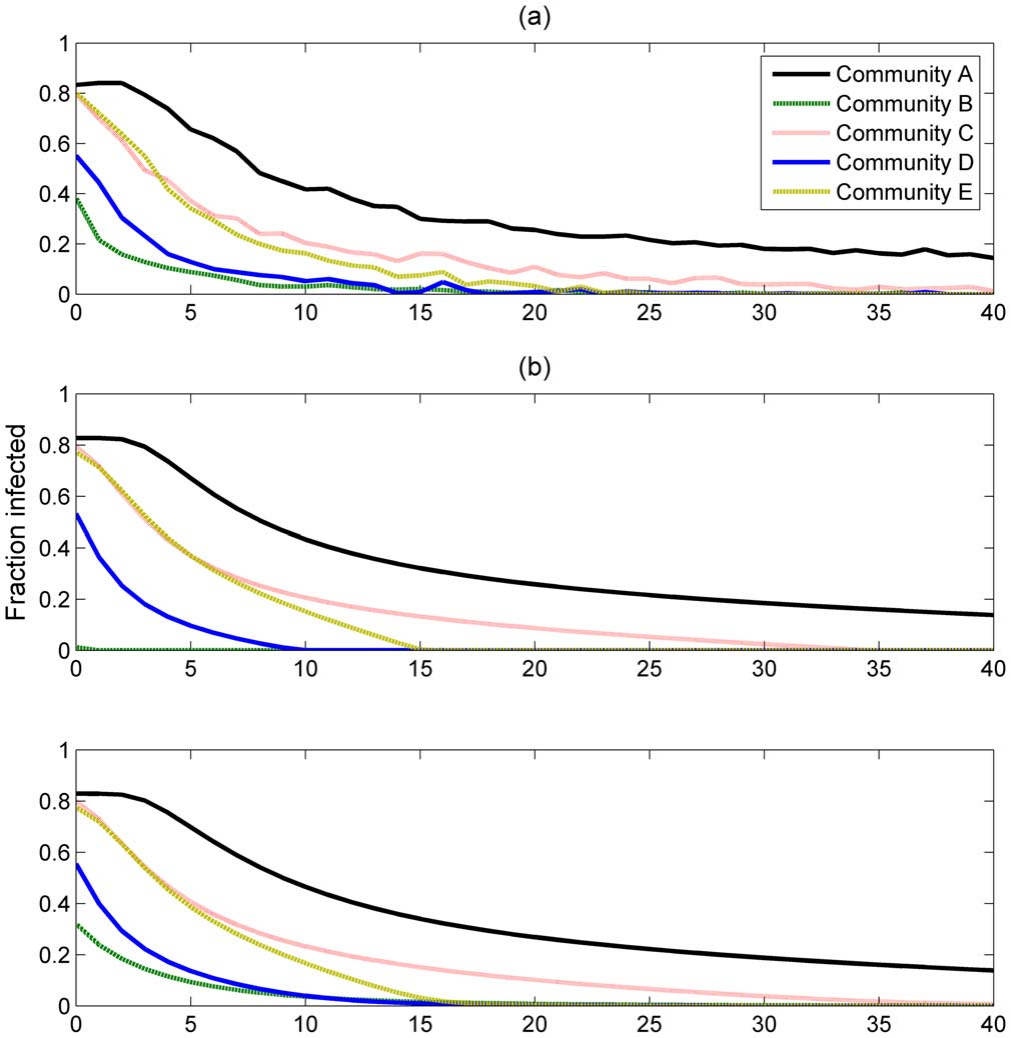
The prevalence of the disease within a community, plotted as a function of J. (a) - results of the individual based simulations, (b) - the isolated mean field approximation, (c) - mean field approximation including boundary node effects. *τ* = 1, *N* = 250 000.

In Figure 3 we plot the average prevalence of the disease in each community (averaged over a large period of time), which is representative of the per-community endemic steady state, over increasing values of *J*. Figure 3 (a) shows the average results of the individual-based simulations of the SIS model. Note that the different communities have a different level of infection even in the case where *J = 0*, due to their different levels of community awareness *H* to the disease. Figure 3 (b) shows the mean field estimates for the isolated community case, calculated as described earlier. Results from both the simulations and these mean field estimates are in close agreement, with the exception of community B. Differences between simulation results and the mean field model are to be expected to some extent, because the mean field is only an approximation which does not take into account an underlying network structure. A small amount of difference can also be attributed to 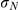: the white noise parameter introduced earlier to model varying awareness levels between members of the same community. This white noise affects the simulations but has a mean of 0 and is therefore not taken into account by the mean field model.

The results of using mean field equation (8), which takes into account the external force of infection, are shown in Figure 3 (c). We note that most communities' levels of infection are largely unaffected: a difference is only visible for community B; with very small differences also notable for community D.

#### The SIR Model

**Figure 4:**
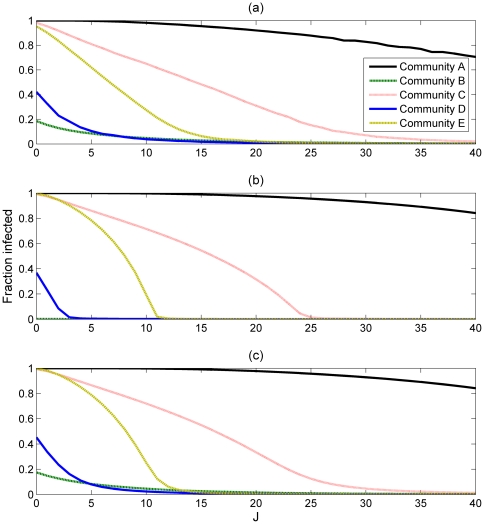
The final size of the epidemic as a fraction of community population, plotted as a function of J. (a) - results of the individual based simulations, (b) - the isolated mean field approximation, (c) - mean field approximation including boundary node effects. *τ* = 0.5, *N* = 250 000.

Figure 4 shows the results for the SIR model, plotting the final size of the epidemic over parameter *J*. When the external transmissions are introduced we observe a large increase in the final size of the epidemic in community B, similar to the results of the SIS model. However, unlike the SIS model, we also observe a noticeable effect in communities C, D and (although to a lesser extent) E (Figure 4 (b) and (c)). In these communities an epidemic occurs for larger values of *J* than in the case where the communities are isolated, and as a result the mean field estimates are closer to the results obtained from the simulations (Figure 4 (a)) than the mean field estimates of the isolated case. The size of the epidemic seems to remain identical in Community A in both mean field approximations. Due to the community's low awareness an infection spreads quickly through its population and any effect of infection being transmitted from the outside is negligible. As observed in the next section, community A is also the origin of the largest number of external transmissions than any other community.

### Model Simulations

#### Intra- and Inter-community Transmissions: Communities without risk perception

**Figure 5:**
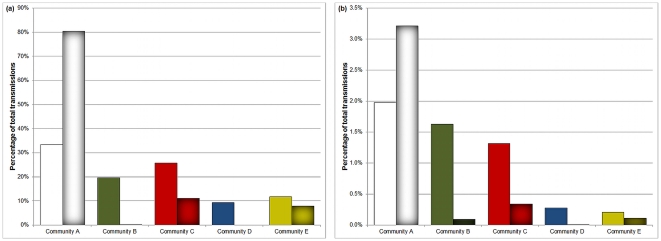
Percentage of transmissions by originating community. (a) shows all transmissions, (b) shows only inter-community transmissions. Non-shaded bars correspond to the case where H = 0 across all communities, shaded bars correspond to H set to the value suggested in Table 1. *τ* = 0.1, *N* = 250 000.

Considering the simple case of no awareness on the network, we study the number of transmissions occurring in each community, identifying which of these transmissions occur between communities, or, in other words, how often transmissions arrive from external groups. The results are shown, by origin of the transmission, in Figure 5 (non-shaded bars). Without any risk perception on the network, the disease spreads with even probability on all edges and the number of total transmissions is roughly proportionally divided between all communities according to their population size. In addition, by examining the results in (b), we see that the number of external infections originating at each community is also roughly proportional to the 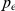 of the community.

#### Intra- and Inter-community Transmissions: Communities with varying levels of risk perception

Risk perception is introduced in the network by setting the *H* value for each community as specified in Table 1. We run the same experiment as before, with the results displayed in the shaded bars of Figure 5. In this set of results the role of the high-awareness communities B and D in spreading the disease decreases dramatically, while A's contribution increases to over 80%. The low external connectivity and high awareness of D have isolated the community from the disease: D has very few external and internal transmissions, suggesting that the community is mostly healthy. Surprisingly, despite its low 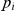, 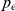 and 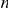 values, community E still accounts for over 5% of total transmissions.

Following the introduction of awareness, the vast majority of transmissions originating in community B are external. Because of the high awareness within B the disease does not transmit within the community, but can still reach groups of lower risk perception, in particular community A (where *H* is still 0). The results shown in Figure 5 were consistent for both the SIS and SIR model, for *τ* = 0.1.

#### Sensitivity to disease infectivity

To assess the sensitivity of the above results to changes in the disease infectivity *τ*, we examine the number of transmissions originating from each community as a function of *τ* for both the SIS and SIR models. We take into account both the cases of no awareness and per-community risk perception as defined in Table 1.

**Figure 6:**
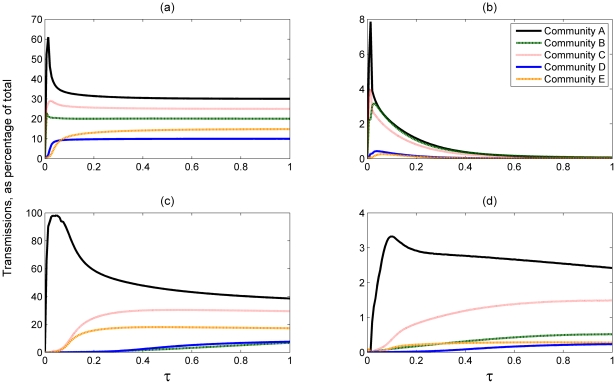
Percentage of transmissions as a function of ??? for each community for the SIS model. (a): Total transmissions, no awareness. (b): External transmissions only, no awareness. (c) and (d) Total and external transmissions respectively, with awareness as specified in Table 1. *N* = 250 000.

When examining the share of the total number of transmissions for each community in the SIS model (in Figure 6 (a) and (c)) we observe that no significant changes occur for values of 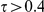, because for larger *τ* the disease is quickly introduced to the whole network. As a result the role of the boundary nodes decreases and the number of external transmissions in the no awareness case (b) decays rapidly with increasing *τ*. This decay is not seen in Figure 6 (d), because the high risk perception levels of some communities guarantee that there will be enough susceptibles in each community for external transmissions to occur. In the case of no awareness, (a), the percentage of transmissions for each community is roughly proportional to the community size, as seen previously. This result is only subject to change for very low *τ*, when community E's number of transmissions is less than D's: as infectivity increases E overtakes D due to its greater size, despite its lower connectivity.

**Figure 7:**
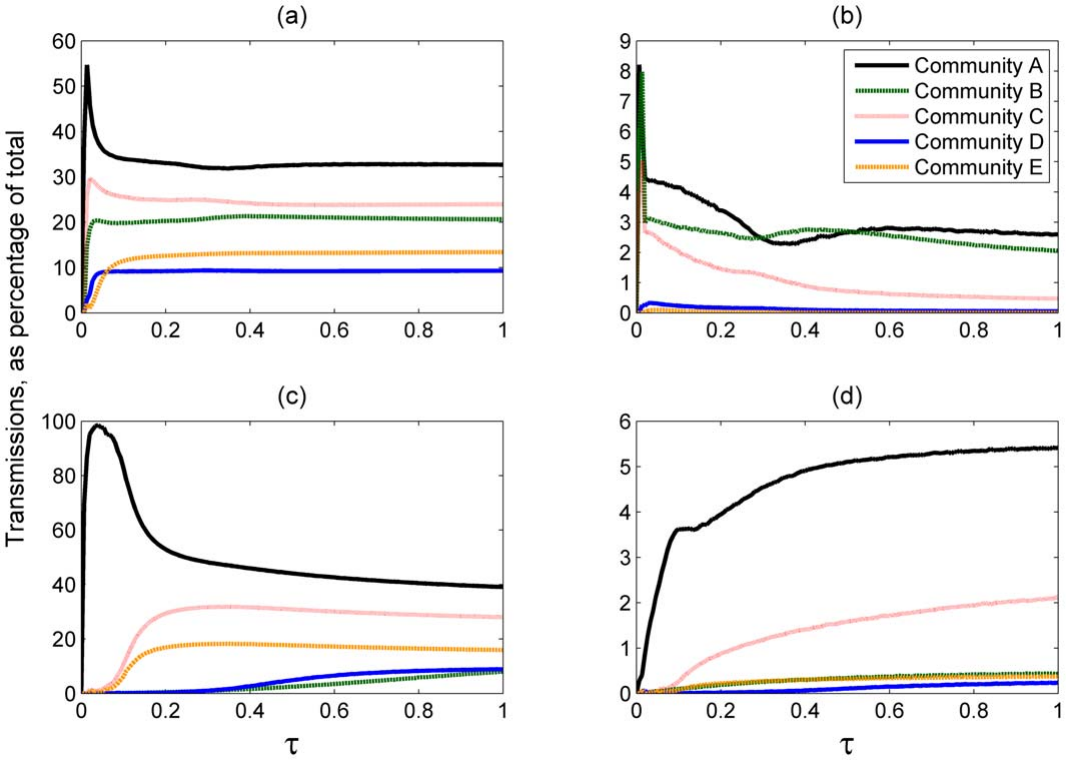
Percentage of transmissions as a function of ??? for each community for the SIR model. (a): Total transmissions, no awareness. (b): External transmissions only, no awareness. (c) and (d) Total and external transmissions respectively, with awareness as specified in Table 1. *N* = 250 000.

The percentage of all transmissions originating from each community and their relationship to *τ*, in both the no awareness and the varying per-community awareness cases (shown in Figure 7 (a) and (c) respectively), is nearly identical for the SIR and SIS models. A notable difference can be seen, however, in the percentage of external transmissions and their dynamics in relation to *τ*. In the no awareness case (Figure 7 (b)) we no longer see the decay in the number of external transmissions originating from communities A and B that was observed for the SIS model. At 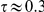 the number of external transmissions originating from community B exceed those of A, although at higher *τ* values the external transmissions of A are once again higher than B's. Another difference between the transmission models is that, for the SIR model, we observe an increase in the number of external infections originating from community A as *τ* increases, unlike the SIS model where the number of external transmissions is decreasing with *τ*.

#### Disease Prevalence

**Figure 8:**
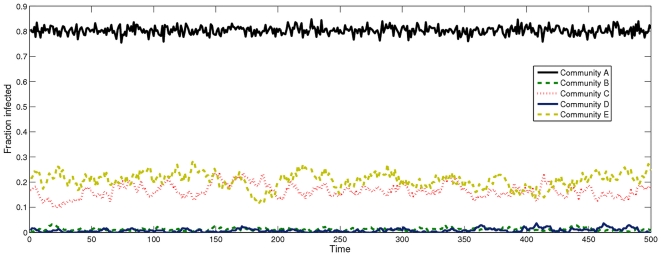
Fraction of infected individuals per community over a window of 500 steps. *τ* = 0.1, *N* = 500 000.

To examine the prevalence of the disease in each community, for simplicity, we look at a time window of 500 time steps, shown in Figure 8. Since the transmission model is SIS and the disease is endemic, the number of infected individuals in each community tends to oscillate around a value which is representative of the level of infection in that community. We can immediately see from the results that the high awareness communities B and D have very low disease prevalence, with the infection even becoming temporarily extinct on several occasions. The level of infection for community A is very high, as expected due to the lack of awareness. Figure 8 also demonstrates the point raised in the Methods section that the current risk perception framework may be unsuitable for an endemic disease: in particular, it is difficult to assume that community A, with a prevalence of 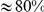, has no awareness whatsoever of the infectious agent. Although the SIS model allows us to examine the endemic steady state of the disease in a network of individuals of various awareness levels, in a realistic setting this risk perception framework may be an unsuitable representation of the actual awareness to an endemic disease.

**Figure 9:**
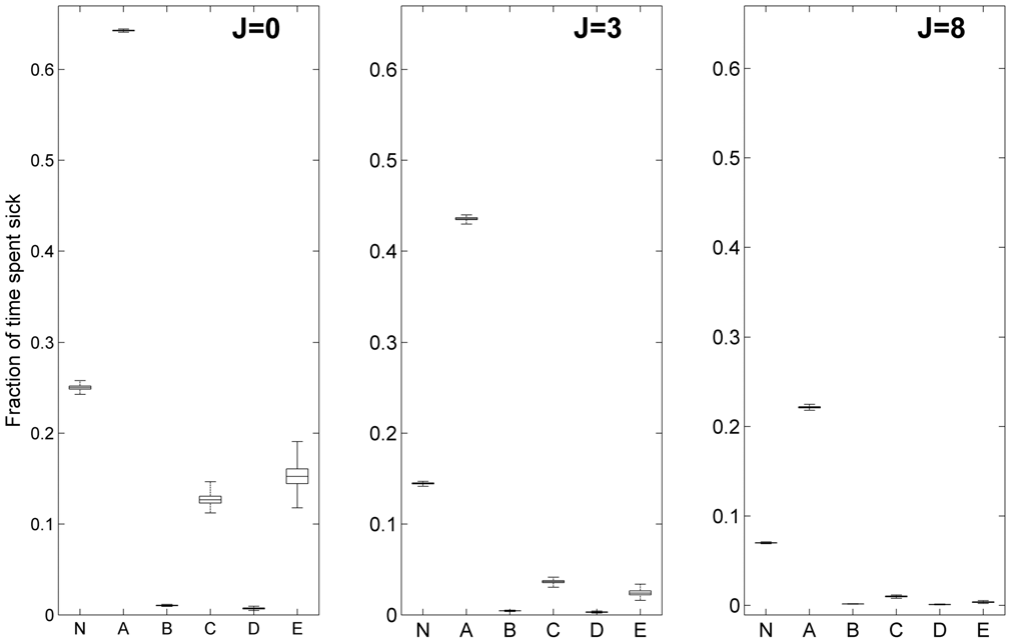
Boxplots of fraction of time spent infected for the whole population (N) and each community. Replicated for three different J values. *τ* = 0.1, N = 250 000.

In addition to the time series window we also examine the average time spent infected for each of the communities and the population as a whole. We also introduce the *J* parameter to confirm the effect of connectivity on risk perception. As we increase *J* we notice that the impact on community E is much greater than on C, supporting the theory that incorporating risk perception has a greater effect on less connected networks. The inter-quartile range for the box plots in Figure 9 is very low, suggesting that the simulation results were in close agreement: over long simulations the average time spent ill for the community converges to a similar value, due to the disease being in endemic steady state. The differences between network topologies are also not that substantial because Erdös-Rényi random graphs have little variation in connectivity between nodes. Figure 9, for the case *J = 0*, supports the results gathered so far in terms of the prevalence in each community.

### Example Applications

#### Modelling Interventions

In this section we draw attention to the similarity between the modelling of mitigation strategies in literature and the risk perception framework. The aim of interventions is to reduce the disease's basic reproductive number 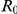 to a lower effective reproductive number 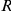 [17]. If the effective reproductive number is less than one then the disease will become extinct, according to the threshold property stated earlier, otherwise the infectious agent still has the potential to spread. We can model the community-wide risk perception as being the result of the interventions. To demonstrate with an example, we use the SIR transmission model and introduce a single infective individual in community A. We change the values of *H* for the communities so that

- *H = 2* for community B;
- *H = 1* for C, D and E; and
- *H = 0* for A.

**Figure 10:**
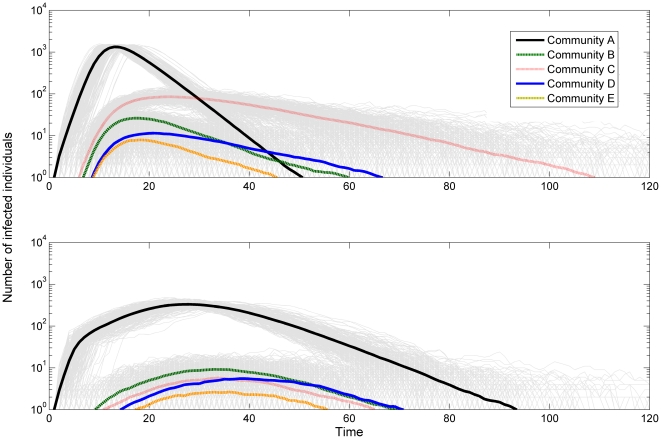
Time series of an epidemic outbreak in the five communities. The lower figure represents the case in which the mitigation strategies, as described in the text, are in place. Light grey lines correspond to results from the separate simulation runs. *τ* = 0.05, N = 250 000.

The size of *H* in this case is used to represent the level of interventions that a community is subjected to: larger *H* means stricter interventions are in place to reduce the disease transmission. Once a number of cases have been observed in the population mitigation strategies are imposed which result in an increase in *H* by one in the large communities A and C. We calculate the mean connectivity of our population and use equation (7) to set 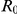 to 2 (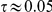), a value applicable for highly-transmissible Influenza [38]. The interventions were applied once 50 infectious cases were registered in a single community. We simulate the disease dynamics both with interventions in place and without, and ignore any simulations where the introduction of the disease fails to cause an epidemic. The simulations begin with a single infectious individual in community A. Averaging the results of the simulation runs we obtain the time series presented in Figure 10. The y-axis, representing the number of cases, is in logarithmic scale, so that even the smaller epidemics appear visible in the Figure. As expected, the epidemic peak in Community A is smaller in the case where interventions are present (Figure 10, bottom) and the duration of the epidemic is longer, which is consistent with a lower *R* value [3]. Due to the interventions in place the epidemic does not spread as effectively to the other communities, resulting in a smaller epidemic size in each of them, with community C, which is itself the subject of interventions, being impacted the most.

Modelling studies have shown that the effectiveness of mitigation strategies can be greatly affected by many factors, such as their timing and duration. There may be little benefit from these strategies if they are applied too late and they might even fail to significantly reduce the size of the epidemic [38]. The work presented here was done simply for illustrative purposes and the details of mitigation strategies and their applications are beyond the scope of this paper.

#### Timing of Outbreaks

In applying mitigation strategies it is also important to detect the outbreak as early as possible. In [39], the authors examine methods for improving the early detection of outbreaks on a social network and propose a strategy that does not require extensive knowledge of the network structure. We propose that community-resolved modelling could similarly be used to determine where to concentrate detection efforts. By modelling a population in terms of communities and simulating an infectious disease outbreak, we may discover that there is a noticeable time period before an outbreak in a particular community reaches the rest of the population. We test this on the artificial population we have studied so far by introducing a single infected in any one of the communities. The model in this case is SIR, and the *H* values of all communities have been set to those specified Table 1. We also estimate the probability of the infection being seeded outside the original community, as opposed to becoming extinct.

We observe that a single case in community A takes on average 7 days to infect at least one other community and that the probability of an infection in A causing an epidemic is very high. The time period is long enough to provide good warning and the high probability of an epidemic justifies applying interventions to prevent the disease spread. On the other hand, most outbreaks in communities D and E are likely to become extinct. The results in Table 2 might not be surprising given our study so far, but modelling other populations could provide interesting case studies. Another interesting observation in these timings is that, for example, it takes roughly 3 days before an epidemic starting at C reaches A, and 13 days before it reaches D. The large difference between these times suggests that most frequently the infection may not travel directly from C to D, but rather pass through an intermediate community, such as A. Such observations may be interesting to epidemiologists attempting to trace the spread of disease.

**Table 2:** Time and probability of an outbreak in a specific community, following a single infected case in one of the other communities.

From	*A*				*B*				*C*				*D*				*E*			
To	*B*	*C*	*D*	*E*	*A*	*C*	*D*	*E*	*A*	*B*	*D*	*E*	*A*	*B*	*C*	*E*	*A*	*B*	*C*	*D*
Time until first case (days)	7.25	5.73	9.77	6.59	3.59	8.37	12.7	9.69	3.41	10.1	12.8	9.45	10.03	16.68	14.07	16.84	10.5	17.2	15.3	19.8
Standard deviation (days)	2.07	1.74	3.56	1.79	2.97	3.67	5.04	3.65	3.23	4.01	4.97	3.84	5.25	6.2	5.66	5.62	6.56	6.74	6.65	7.1
Probability of seeding infection	0.73	0.73	0.7	0.73	0.36	0.35	0.32	0.34	0.48	0.45	0.42	0.45	0.003	0.003	0.003	0.003	0.06	0.05	0.06	0.05

## Discussion

The concept of metapopulations, mentioned in the introduction, has been used extensively in epidemiological studies for dividing a target population spatially into interacting patches [6]. The community based approach is a similar concept, although the division does not have to be geographical, but rather based on frequency of contact. As a result, for example, people working for the same company are likely to be members of the same community as the majority of them will be densely connected from a social network point of view. Furthermore, these individuals may have a similar level of risk perception to the disease, based on word of mouth, company policy, etc. By being agent-based, our community modelling approach is likely to provide more fine grained detail about the spread of the disease from person to person and take into account the particular topology of each community structure in the population. Using community-resolved population modelling we can also examine the metapopulation concepts of persistence, extinction and seeding of infection as it occurs between the communities. Another approach for modelling a real-world population would be the previously mentioned suggestion by Davey and Glass [24]: to construct large populations as a set of smaller interacting communities. This suggestion supports a ‘bottom-up’ approach, where the population is built up by linking the separate communities. The structure of these communities does not have to be precise, and can be estimated as long as it approximates the properties of the population to a reasonable extent. Finally, if a contact network for a population of any size is available or estimated, one can run community detection algorithms to identify the communities present and assign awareness values as necessary.

Our study has concentrated on examining various properties of community-structured networks, as well as risk perception. The interpretations of our results are summarised in the following subsections.

### 

#### Single Community

The results of the single community experiment, visualised in Figure 2, showed that the underlying parameters of the most highly exposed community changed as the community's *H* increased. For all *H*, communities with 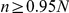 or those with low 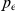 were found to have the least exposure to external infection. This result is not surprising, because in both these cases the number of external connections that the community has is very low: low 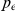 means less connections are formed, whereas if *n* is high there are less external nodes available to connect to. As *H* increases a notable difference is the shift of highest exposure away from large communities with medium to high 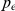 and towards medium sized communities with high external connectivity instead. This phenomenon is caused by the effect of increasing *H* on the probability of becoming infected. Increasing *H* dampens the infectivity by a factor of 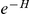 and the resulting reduction in the probability of becoming infected (equation (5)) can only be offset by an increase in 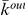. In the case of a large and highly externally connected community there are a large number of boundary nodes with low degrees (because there are less external nodes to connect to). As *H* increases such nodes do not become infected as often and the emphasis shifts to medium sized communities instead. These communities have less boundary nodes which have a much higher degree each, offsetting the reduced effective infectivity. Thus, overall, with increasing *H*, the exposure to infection of a community is influenced less by the number of boundary nodes and instead influenced by these nodes' external connectivity. An example from the multi-community simulations can be observed in the time series of Figure 8, where the average level of infection suffered by communities C and E is almost identical, despite C's awareness being twice higher than E's. In this case, community C is more highly connected both internally and externally than community E, which offsets C's higher *H* parameter.

#### The role of external and internal connectivity

The mean field results for both transmission models (Figures 3 and 4) showed that most communities' levels of infection are largely unchanged by the introduction or removal of the external force of infection to the mean field equations (9). The lack of any considerable change in infection levels for these communities suggests that external connectivity plays a lesser role for a disease which is already established inside a community. A disease spreads more efficiently within a community than across communities because the number of connections between individuals inside the community is greater than the number of external connections. The lower efficiency in spread across communities is supported by the simulations, which show a very small number of inter-community transmissions for all communities both with and without incorporating risk perception (Figure 5). Thus the main contribution of the external connectivity is to reintroduce the disease if necessary and to maintain the infection in communities where its prevalence is low. For high awareness communities, such as B, the disease is unable to circulate for a long time within the community, and the infection has to be continuously re-introduced by the outside population: as a result the prevalence of the disease in community B increases substantially when we consider the community's external connections.

Internal connectivity also plays a role in a community's efficiency in transmitting the infection to the outside world. In Figure 5, Community A is responsible for the largest number of external transmissions, despite having similar size and lower or equal external connectivity in comparison to communities B and C. The reason behind the large number of external transmissions is that the community's higher internal connectivity allows the infection to reach the boundary nodes faster than in other communities, in order for the disease to reach the rest of the network. Further evidence can be seen when examining the number of external transmissions as *τ* varies. In Figure 7 (b) (i.e. SIR model, no awareness), for a small range of *τ* values, community B's external transmissions exceed those of A, likely due to the former community's higher external connectivity. However, for higher *τ*, due to A's larger size and higher internal connectivity the disease propagates to the boundary nodes faster and is able to infect external nodes before any of the other communities. This effect is not observed for the SIS model, because nodes do not recover and hence the disease can continuously spread between the communities, reducing the importance of community A being the most efficient spreader. From the above considerations of the disease dynamics of community A we can conclude that of importance to the population-wide spread of infection is not only the externally connectivity of a community, but also the efficiency with which the disease propagates within it via the internal connections.

#### The role of boundary nodes in the SIS and SIR transmission models

The difference between the role of the boundary nodes in the SIS and SIR models is that in the former boundary nodes can transmit the disease repeatedly, since they become susceptible again following infection. In contrast, in the SIR model, upon recovery a boundary node can no longer export or import the infection. The argument presented in the previous subsection was that the boundary nodes serve the minor role of introducing the disease inside the community after which the disease spreads more efficiently over the more numerous internal links. In the SIR model however each node is only infectious once, after which it recovers permanently, meaning that the number of infections that may occur inside a community is bounded. As a consequence, once the disease has infected a community, the number of internal transmissions does not grow unboundedly to vastly outstrip the number of external ones and the difference between the total number of internal and external transmissions is reduced. Thus, in the SIR model without risk perception (Figure 7 (b)), we no longer observe the aforementioned decaying effect in the number of communities A and B's external transmissions for increasing *τ*, due to these communities' high external exposure. This effect, a rapid decrease in the number of external transmissions with increasing *τ*, was initially observed in the SIS model without risk perception presented in Figure 6 (b).

In the equivalent risk perception simulations we note a further difference between the external transmissions of community A in the SIR (Figure 7 (d)) and the SIS models (Figure 6 (d)). Namely, we observe that the number of external transmissions for A increase with *τ* in the SIR model, whereas they are seen to increase initially and then slowly decay for the SIS model. If the boundary nodes do not become susceptible again following infection, for increasing *τ*, community A is able to spread the disease to other communities at a high rate. These communities will, in turn, have a reduced probability of transmitting the disease to external acquaintances as the no-awareness community A's boundary nodes would be recovered. Thus, due to becoming immune to the disease after infection, the boundary nodes prevent the disease from re-entering their community.

The above comparison of the SIS and SIR model, provided by the results in Figures 6 and 7, demonstrates that boundary nodes have a greater impact on the spread of infection in the SIR than in the SIS model. Such a conclusion is also supported by the overall impact of introducing the external force of infection to the mean field of both transmission models (Figures 3 and 4): the SIR results displayed a greater increase in infection levels following the introduction.

#### The role of Community A in the presence of risk perception

The high internal and external numbers of transmissions observed in A (cf. Figure 5) imply that people who take no precautions to reduce their susceptibility are a danger not only to themselves, but also to other groups of higher risk perception as well. A's inter-community transmissions represent the largest amount compared to all other groups and the difference becomes even more expressed when awareness is introduced. When all inter-community links are removed, so that the communities are isolated, the disease becomes extinct in all communities except A, demonstrating A's vital role in maintaining the infection. In a real world context this result supports the idea that concentrating on ‘high risk’ groups when providing vaccines and other preventative measures will be of great benefit to the rest of the population as well. From an economic perspective, the external transmissions originating from community A can be described as a significant cost to the whole population [31].

When examining the prevalence of the disease in each community (Figure 8) or the fraction of time members of each community spend in the infected state (see Figure 9), we also note that averaging across the whole population does not provide a representative measure of the disease prevalence or the time spent sick in each community: e.g. all communities except A spent less time sick than the average for the population. This illustrates how dividing the population into communities can help in identifying the social groups which suffer most of the burden of the disease.

In addition to the above results, in this manuscript we have given examples of applications of community-resolved modelling, including using the awareness framework to model interventions and to approximate the timing of outbreaks as the disease spreads through the communities that form the population of interest. Despite being based on a synthetic network, rather than on real-world data, the work presented in this manuscript could still contribute to the existing literature on epidemiological modelling by introducing community-structured networks as a potential contact network model and describing some of the benefits of modelling a target population as a set of interacting heterogeneous groups.

#### Conclusion

In this paper we examined the process of disease transmission on a theoretical population consisting of heterogeneous communities. The spread of infectious disease has not been studied and characterised on idealised community-structured graphs, as it has been on lattices or networks with small-world and scale-free properties [8]. By considering a theoretical model we have demonstrated how the differences between the communities' properties could affect the disease dynamics. In particular we have examined how often infections can reach certain communities and the role of boundary nodes in the transmission process. We have provided mathematical approximations in addition to the agent-based model. While observing the results we noticed that the communities' properties also determine whether the disease will persist locally or become extinct and how the infection is seeded between communities. The approach we used to generate our theoretical population is also novel, although based on the existing l-planted partition algorithm [34].

By introducing our concept of risk perception into our model we allowed to further differentiate between the communities and take into account how varying levels of risk averseness to infection can reduce the size of the outbreak in some communities. Doing so allowed us to demonstrate that communities with little or no awareness to the disease can still play a vital role in maintaining the infection even in the case where all other communities act, based on their perceived risk, to reduce their exposure to the disease.

The model presented here is purely theoretical, although we do provide a discussion of potential applications and implementations. We have discussed possible methods for modelling a real population using both real data and approximations on the community level and examined how the risk perception framework could be used to generalise the level of intervention present in a community. By considering the time it takes for an infection in a community to spread to the rest of the population, we also suggest that outbreak detection can be concentrated on a particular community.

The work presented demonstrates some of the advantages of using a community resolved approach to modelling in epidemiology. One possible direction for future work is to consider the concept of overlapping communities [22], where a single individual is a member of multiple communities. To represent social distancing and similar measures taken to prevent exposure to infection, we can also consider dynamic networks, in which the edges between individuals vary with time. Any further work should aim to expand our knowledge of the effect of community structure on the spread of disease. Applying this approach to real world data would also allow us to better evaluate its practical uses.
